# Triterpenoid saponin from *Panax ginseng* increases the sensitivity of methicillin-resistant *Staphylococcus aureus* to β-lactam and aminoglycoside antibiotics

**DOI:** 10.1128/spectrum.03227-23

**Published:** 2024-04-22

**Authors:** Sakura Tsutamoto, Yuina Iwasaki, Akari Shinohara, Risa Imamiya, Keiichi Samukawa, Miki Kawada-Matsuo, Hitoshi Komatsuzawa, Yui Yamada, Kouki Mandokoro, Hiroshi Iwao, Yasuhiko Horiguchi, Mayuko Osada-Oka

**Affiliations:** 1 Food Hygiene and Environmental Health, Division of Applied Life Science, Graduate School of Life and Environmental Sciences, Kyoto Prefectural University, Kyoto, Japan; 2 Food Hygiene and Environmental Health, Faculty of Life and Environmental Sciences, Kyoto Prefectural University, Kyoto, Japan; 3 Department of Pharmacology, Graduate School of Medicine, Osaka Metropolitan University, Osaka, Japan; 4 Department of Bacteriology, Graduate School of Biomedical and Health Sciences, Hiroshima University, Hiroshima, Japan; 5 Kyoto Prefectural Chutan Livestock Health Hygiene, Fukuchiyama, Japan; 6 Department of Molecular Bacteriology, Research Institute of Microbial Diseases, Osaka University, Suita, Japan; JMI Laboratories, North Liberty, Iowa, USA

**Keywords:** *S. aureus*, multidrug resistance, triterpenoid, *Panax ginseng*

## Abstract

The triterpenoid saponins, ginsenosides, are the major bioactive compound of red ginseng and can exert various physiological activities. In the present study, we examined whether red ginseng extract (RGE) exerts antibacterial activity against methicillin-resistant *Staphylococcus aureus* (MRSA). RGE had no bactericidal activity, at least in the range of dissolvable concentration. However, RGE reduced 0.03–0.25-fold the minimum inhibitory concentration (MIC) values of β-lactam antibiotics (oxacillin, ampicillin, carbenicillin, and cefazolin) and aminoglycoside antibiotics (kanamycin and gentamicin) against the two laboratory strains of MRSA. Moreover, the fractional inhibitory concentration index indicated the synergistic activity of RGE with each of the antibiotics. RGE also increased the kanamycin sensitivity of 15 MRSA strains isolated from human volunteers and increased the ampicillin sensitivity of five MRSA strains isolated from dairy cows diagnosed with bovine mastitis. In contrast, RGE did not alter the MIC values of fosfomycin, tetracycline, and erythromycin, suggesting that RGE acts selectively. In contrast, Triton X-100, which was reported to reduce the MIC value of β-lactam antibiotics to MRSA by increasing membrane permeability, reduced the MIC values of fosfomycin and tetracycline. These results indicate that RGE increases the bactericidal effect of antibiotics via a mechanism different from that used by Triton X-100. We found that ginsenoside Rg3 (Rg3), a component of RGE, was an essential compound that exhibits synergy activity with antibiotics. Furthermore, the non-natural compound K, which contains a common protopanaxadiol aglycon moiety with Rg3, also showed synergistic activity with antibiotics. Thus, Rg3 and compound K are potentially new antibiotic adjuvants against MRSA.

**IMPORTANCE:**

Methicillin-resistant *Staphylococcus aureus* (MRSA) is a multidrug-resistant organism that is prevalent worldwide. Therefore, the research and development of new agents against MRSA are required. We first found that ginsenoside Rg3 (Rg3) in red ginseng, made from the roots of *Panax ginseng* C. A. Meyer, increased the sensitivity of β-lactam antibiotics and aminoglycoside antibiotics to MRSA. Furthermore, we identified that compound K, an unnatural ginsenoside analog, also increased the sensitivity of antibiotics to MRSA, similar to Rg3. By contrast, neither Rg3 nor compound K increased the sensitivity of fosfomycin, tetracycline, and erythromycin to MRSA, suggesting that these act selectively. In the present study, the natural compound Rg3 and its structural isomer, compound K, are potentially new antibiotic adjuvants against MRSA. Currently, multiple antibiotics are used to treat MRSA, but the use of these adjuvants is expected to enable the treatment of MRSA with a single antibiotic and low concentrations of antibiotics.

## INTRODUCTION

Multidrug-resistant *Staphylococcus aureus*, commonly referred to as methicillin-resistant *S. aureus* (MRSA), is a prominent antimicrobial resistance among human pathogens, with the World Health Organization reporting a marked increase. MRSA is predominantly a nosocomial pathogen that causes hospital-acquired infections; however, it is now commonly isolated from individuals with community-acquired infections. Currently, healthcare-associated infections are more likely to be caused by community-acquired MRSA (CA-MRSA) strains than hospital-associated MRSA (HA-MRSA) strains (1). The spread of MRSA strains has stimulated the search for new strategies to treat these multidrug-resistant pathogens. In recent years, the search for novel products with antibacterial activity has become increasingly important alongside the discovery of new synthetic chemical compounds with antibiotic properties (2). It is thought that the solution to emerging antibiotic resistance might involve a combination therapy of existing antibiotics and potentiating adjuvants, given the current stagnation of new antimicrobial drug development. Komatsuzawa et al. (3, 4) reported that non-ionic detergent, Triton X-100 enhanced the susceptibility of MRSA to β-lactam antibiotics by the enhancement of lipoteichoic acid (LTA) release. LTA is implicated in the triggering of bacterial autolysin activity so that the bacteriolytic and bactericidal effects of β-lactam antibiotics are brought about. Thus, compounds that promote the release of cellular LTA are candidates for antibiotic adjuvants.

Red ginseng, made from the roots of *Panax ginseng* C. A. Meyer, is a source of many phytochemicals with therapeutic potential and has been used as a traditional medicine to treat various diseases for more than 2,000 years (5, 6). Ginsenosides are a class of natural product steroid glycosides and triterpene saponins responsible for the pharmacological effects of red ginseng (7–10). Previously, we reported that ginsenosides inhibit the phosphorylation of p70 ribosomal S6 kinase in the ears of mice with atopic dermatitis (AD), resulting in the suppression of skin inflammation (11). While *S. aureus* is a commensal bacterium that colonizes the skin of one-third of humans, it is a major pathogen for patients with AD. Therefore, we analyzed whether red ginseng extract (RGE) exhibits antibacterial action. Nearly 20 ginsenosides have been identified in red ginseng, and they are classified as either major ginsenosides (>80% of the total ginsenosides, e.g., Rb1, Rg1, and Re) or minor ginsenosides (e.g., Rg3 and Rh1) (10, 12). The major ginsenosides are present in the raw roots of *Panax ginseng*. In contrast, the minor ginsenosides are usually found in heat-processed ginseng products such as red ginseng. Moreover, most of the identified ginsenosides are further classified into three groups based on the structure of the aglycone moieties: 20S-protopananxadiol (PPD) type (Rb1 and Rg3), 20S-protopananxatriol (PPT) type (Rg1, Re, and Rh1), and oleanolic acid. Compound K, which is reported as a biologically active substance, is detected inside the human body as a metabolite of Rb1 (8). Compound K also consists of PPD aglycon. In the present study, we examined the antibacterial activity of RGE, five ginsenosides, and compound K against MRSA.

## RESULTS

### RGE reduces the MIC of antibiotics against MRSA

**Fig 1:**
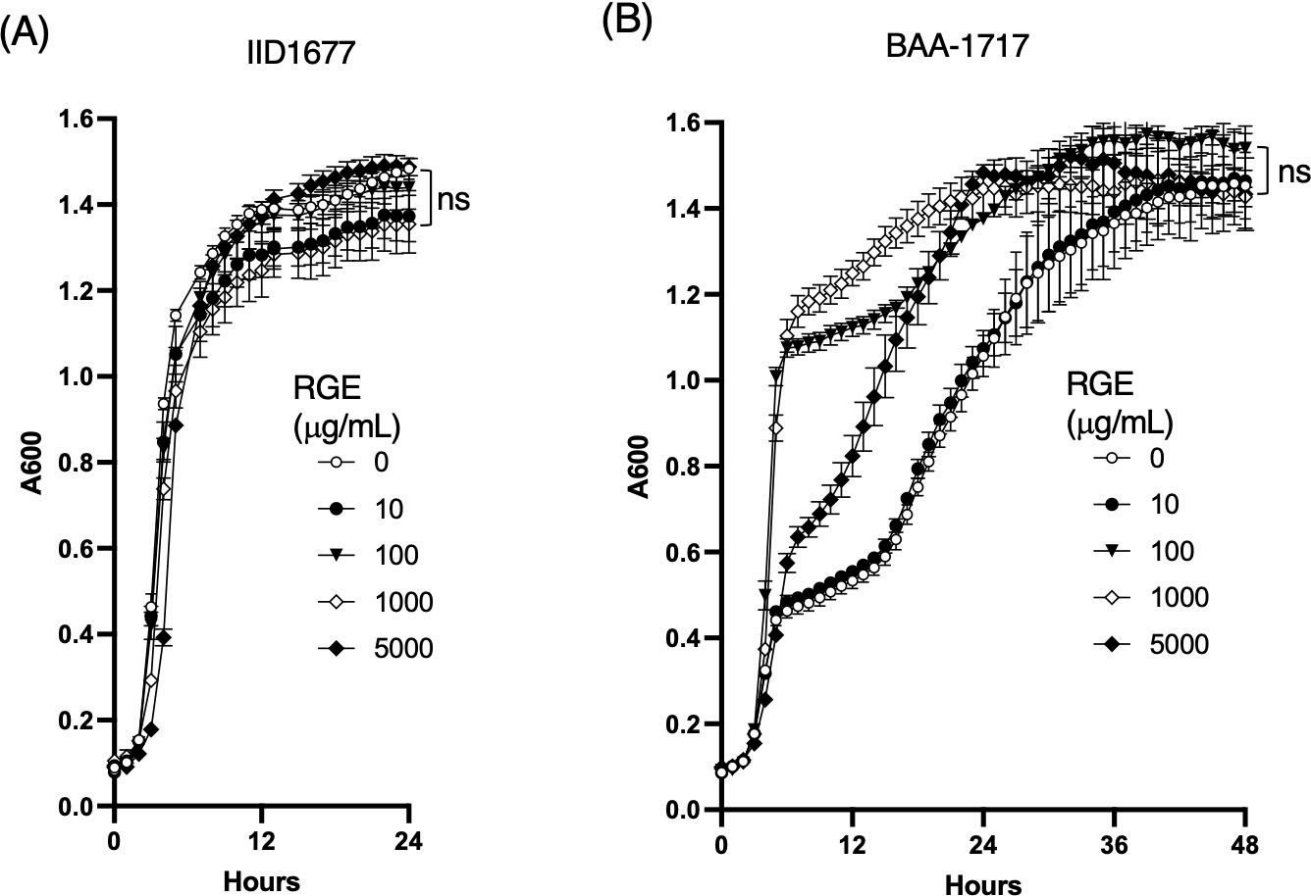
The growth curve of MRSA strains in the presence of red ginseng extract. The growth of the bacteria (IID1677 and BAA-1717 strains) was monitored with absorbance at 600 nm (A600) for 24–48 h at 37°C in the absence or presence of RGE (10–5,000 microg/mL) (**A**) and RGE (5,000 microg/mL) (**B**). Before the measurement of absorbance, the plate was shaken for 5 s. One hundred microliters of suspension containing bacteria in brain heart infusion broth (OD_600_ = 0.00045) and RGE at the indicated concentrations was added per well of a 96-well plate. Values are expressed as the mean ± SD (*n* = 3) of at least three independent biological replicates. Two-way ANOVA and Tukey’s test were used for statistical analysis; ns, not significant.

First, we investigated whether RGE inhibits the growth of MRSA strains IID1677 and BAA-1717. After being cultured with RGE (10–5,000 µg/mL) for 24 h, the growth of MRSA IID1677 was the same as the growth without RGE (Fig. 1). However, although the growth of BAA-1717 was unchanged at 10 microg/mL, higher concentrations of RGE (100 and 5,000 µg/mL) stimulated the early growth of this strain. The bacterial cell densities were similar after 30 h, regardless of the dose of RGE; thus, we concluded that at least RGE had no bactericidal effect, although RGE slightly increased the growth of the BAA-1717 strain.

MRSA IID1677 and BAA-1717 were resistant to oxacillin, ampicillin, carbenicillin, cefazolin, fosfomycin, kanamycin, gentamicin, tetracycline, and erythromycin antibiotics (Table 1). However, both were sensitive to levofloxacin and vancomycin. Strain IID1677 was resistant to tetracycline, whereas BAA-1717 was susceptible. Next, we examined the effects of RGE on the sensitivity of MRSA strains to β-lactam antibiotics. For strain IID1677, RGE (1,000 µg/mL) reduced the minimum inhibitory concentration (MIC) value for oxacillin from 62.5 to 1.95 μg/mL, and the fractional inhibitory concentration (FIC) index was 0.13, indicating the synergistic activity of RGE with oxacillin (Table 2). The MIC value of oxacillin with 5,000 µg/mL RGE was the same as that with 1,000 µg/mL RGE, suggesting that the efficacy of RGE was limited. For strain BAA-1717, the MIC value of oxacillin decreased by one-fourth and one-eighth in the presence of RGE (100 and 1,000 µg/mL), and RGE above 100 µg/mL showed synergistic activity with oxacillin (Table 3). Furthermore, RGE (100 and 1,000 µg/mL) increased the sensitivity of these bacteria to other β-lactam antibiotics (i.e., ampicillin, carbenicillin, and cefazolin) (Tables 2 and 3).

**TABLE 1:** MIC of antibiotics against methicillin-sensitive *S. aureus* (MSSA) or MRSA^*a*^

Antibiotic class	Antibiotics	MSSA	MRSA	
		JCM2151	IID1677	BAA-1717
Penicillin β-lactam	Oxacillin	<3.91	62.5	62.5
	Ampicillin	<7.81	2,000	2,000
	Carbenicillin	<3.91	250–500	125–250
Cephalosporin	Cefazolin	<7.81	125	62.5–125
Phosphonic	Fosfomycin	50	200	400
Aminoglycoside	Kanamycin	<78	5,000–10,000	>80,000
	Gentamicin	<0.156	2.5	10
Tetracycline	Tetracycline	<1.56	200	<1.56
Macrolide	Erythromycin	<1.95	>500	31.3
New quinolone	Levofloxacin	0.156	0.156–0.313	0.313–0.625
Glycopeptide	Vancomycin	1.25	1.25	2.5

^*a*^ Boxes shaded in gray are estimated as sensitive.

**TABLE 2:** Synergistic combination of antibiotics and RGE against MRSA IID1677 strain^*a*^^,*b*^

Antibiotics	FIC index						FIC index (min)
	Antibiotics + RGE (μg/mL)						Antibiotics + Triton X-100
	0	1	10	100	1,000	5,000	
Oxacillin	1	1	1	0.51	0.13	0.53	0.01
Ampicillin	1	1	1	0.51	0.35	0.75	0.07
Carbenicillin	1	1	1	0.51	0.35	0.75	0.02
Cefazolin	1	1	1	0.51–1.01	0.35–0.60	0.35–0.60	0.01
Fosfomycin	1	1	1	1.01	1.10	1.50	0.14
Kanamycin	1	1	0.5	0.14	0.16	0.56	0.14
Gentamicin	1	1	0.5	0.26	0.23	0.63	0.26
Tetracycline	1	1	1	0.51	0.60	1	0.26

^*a*^ The FIC index analysis for the combination of antibiotics and each concentration of RGE (1–5000 microg/mL). The FIC index minimum (min) analysis for the combination of antibiotics and Triton X-100 (0.0025%–0.002%).

^*b*^ Boxes shaded in gray are estimated as synergy.

**TABLE 3:** Synergistic combination of antibiotics and RGE against MRSA BAA-1717 strain^*a*^^,^^*b*^

Antibiotics	FIC index						FIC index (min)
	Antibiotics + RGE (μg/mL)						Antibiotics + Triton X-100
	0	1	10	100	1,000	5,000	
Oxacillin	1	1	1	0.26	0.23	0.63	0.03
Ampicillin	1	1	1	0.51	0.35	0.75	0.07
Carbenicillin	1	1	1	1.01	0.60	1	0.07
Cefazolin	1	1	1	0.51	0.35	0.75	0.01
Fosfomycin	1	1	1	1.01	1.10	1.50	0.04
Kanamycin	1	1	1	1.01	0.16	0.56	0.1
Gentamicin	1	1	1	0.51	0.23	0.63	0.3
Erythromycin	1	1	1	1.01	1.10	1.50	1.00

^*a*^ The FIC index analysis for the combination of antibiotics and each concentration of RGE (1–5,000 microg/mL). The FIC index minimum (min) analysis for the combination of antibiotics and Triton X-100 (0.0025%–0.002%).

^*b*^ Boxes shaded in gray are estimated as synergy.

Next, we examined whether RGE affects the sensitivity of bacteria to aminoglycoside antibiotics. For IID1677, the MIC value of kanamycin fell by one-eighth and one-sixteenth in the presence of RGE (100 µg/mL and 1,000 µg/mL), and their FIC index were 0.14 and 0.16, indicating the synergistic activity of RGE with kanamycin (Table 2). For the BAA-1717 strain, RGE (1,000 µg/mL) also reduced the MIC value of kanamycin from >80,000 to 5,000 µg/mL, and the FIC index was 0.16, indicating the synergistic activity of RGE with kanamycin (Table 3). Furthermore, RGE (100 and 1,000 µg/mL) increased the sensitivity of these bacteria to other aminoglycoside antibiotics, e.g., gentamicin (Tables 2 and 3). By contrast, the sensitivities of IID1677 and/or BAA-1717 to fosfomycin, tetracycline, and erythromycin remained unchanged in the presence of RGE. RGE (1,000 µg/mL) also increased the sensitivity of kanamycin to 15 of 17 human isolates (Table 4) and that of ampicillin to five of seven bovine mastitis isolates (Table 5).

**TABLE 4:** Synergistic combination of antibiotics and RGE against the CA-MRSA^*a*^^,^^*b*^

Strain no.	MIC (μg/mL)		FIC index^*a*^
	Kanamycin	Kanamycin + RGE	
1	625	156	0.45
2	2,500	313	0.33
3	1,250	625	0.70
4	39.1	9.77	0.45
5	19.5	4.88	0.45
6	39.1	4.88	0.32
7	19.5	4.88	0.45
8	10,000	625	0.26
9	39.1	4.88	0.32
10	39.1	4.88	0.32
11	39.1	9.77	0.45
12	78.1	9.77	0.33
13	4.88	2.44	0.70
14	78.1	9.77	0.33
15	39.1	2.44	0.26
16	39.1	4.88	0.32
17	19.5	4.88	0.45

^*a*^ The FIC index analysis for the combination of kanamycin (0.122–20,000 microg/mL) with each concentration of RGE (1,000 microg/mL).

^*b*^ Boxes shaded in gray are estimated as synergy.

**TABLE 5:** Synergistic combination of antibiotics and RGE against the livestock associated-MRSA^*a*^^,^

Strain no.	MIC (μg/mL)		FIC index
	Ampicillin	Ampicillin + RGE	
1	250	125	0.70
2	250	62.5	0.45
3	7.81	1.95	0.45
4	500	125	0.45
5	500	125	0.45
6	500	250	0.70
7	500	125	0.45

^*a*^ The FIC index analysis for the combination of ampicillin (0.244–1,000 microg/mL) (b) with each concentration of RGE (1,000 microg/mL).

### Effects of Triton X-100 and RGE on sensitivity to antibiotics

The point of action to MRSA by the antibiotics with which RGE exhibited synergistic activity was different between β-lactam and aminoglycoside (Tables 2 and 3). We hypothesized that RGE might have increased the permeability of the bacterial membranes. Triton X-100 increases the sensitivity of MRSA to β-lactam antibiotics by affecting the bacterial membranes when used at less than bactericidal concentrations (13, 14). Therefore, we next compared the effects of Triton X-100 (0.0025%–0.02%) and RGE on the sensitivity of MRSA strains to antibiotics. Triton X-100 (0.0025%–0.005%) has shown synergistic activity with β-lactam (oxacillin, ampicillin, carbenicillin, and cefazolin) antibiotics, and Triton X-100 (0.01%–0.015%) has shown that activity with aminoglycoside (kanamycin and gentamicin) antibiotics (Tables 2 and 3). In addition, Triton X-100 reduced the MIC values of fosfomycin against IID1677 and BAA-1717 and the MIC values of tetracycline against IID1677, but not the MIC values of erythromycin. Thus, the types of antibiotics that exhibit synergistic effects are different between RGE and Triton X-100.

### Rg3 is the bioactive compound in RGE responsible for increasing antibiotic susceptibility

**Fig 2:**
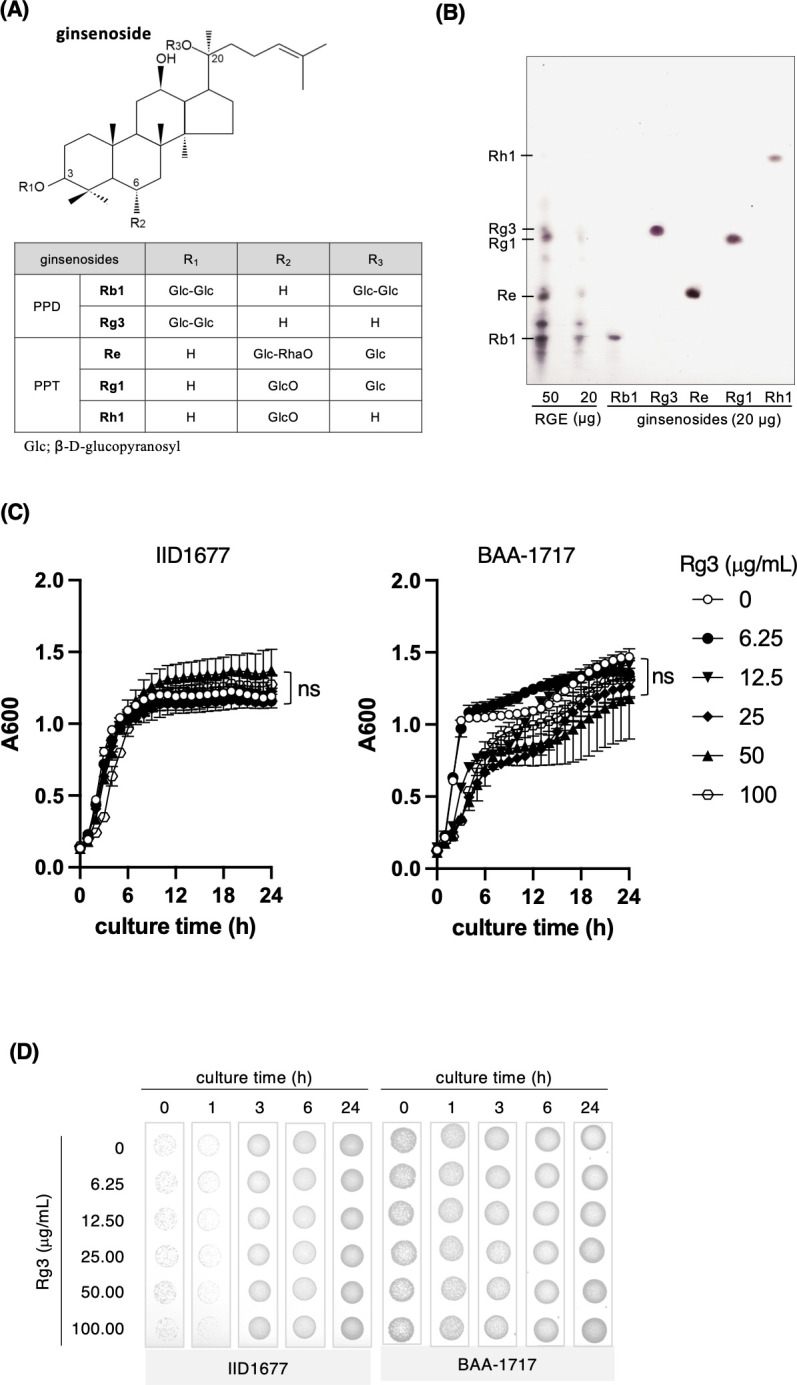
The growth of MRSA strains in the presence of Rg3. (**A**) The structures shown are ginsenosides Rb1 and Rg3, which have the aglycon of 20S-protopananxadiol type and ginsenosides Re, Rg1, and Rh1, which have the aglycon of 20S-protapananxatriol. (**B**) Red ginseng extracts (20 and 50 µg/spot) and five ginsenosides (20 µg/spot) were analyzed by thin layer chromatography. (**C**) The growth of the bacteria (IID1677 and BAA-1717 strains) was monitored with absorbance at 600 nm (A600) for 24 h at 37°C in the absence or presence of Rg3 (6.25–100 µg/mL). Before the measurement of absorbance, the plate was shaken for 5 s. One hundred microliters of suspension containing bacteria suspended in brain heart infusion broth (OD_600_ = 0.00045) and Rg3 at the indicated concentrations was added per well of a 96-well plate. Values are expressed as the mean ± SD (*n* = 3) of at least three independent biological replicates. Two-way ANOVA and Tukey’s test were used for statistical analysis; ns, not significant. (**D**) After incubating the bacteria with each concentration of Rg3 for 0, 1, 3, 6, and 24 h at 37°C in a 96-well plate, a 20-µL aliquot of bacterial culture that was diluted to 10^−2^ was spotted on LB agar. Bacterial growth was visually examined.

Multiple ginsenoside compounds were found in our RGE (Fig. 2A and B); therefore, we tried to identify the specific ginsenoside responsible for increasing the antibiotic sensitivity of MRSA. To do this, we examined the effects of five different ginsenosides (Rb1, Rg3, Re, Rg1, and Rh1) on sensitivity to β-lactam and aminoglycoside antibiotics (Fig. 2A and B). Among the five ginsenosides, only Rg3 (100 µg/mL) reduced the MIC value of β-lactam (oxacillin, ampicillin, carbenicillin, and cefazolin) and aminoglycoside (kanamycin and gentamicin) antibiotics for the IID1677 strain and the BAA-1717 strain and showed synergistic activity, but not the other ginsenosides (Tables 6 and 7). These results indicate that Rg3 has an important role in the increased sensitivity of MRSA to β-lactams and aminoglycosides. Rg3 (6.25–100 µg/mL) did not affect the growth of the IID1677 strain (Fig. 2C and D). Rg3 (25–100 μg/mL) slightly decreased the growth of the BAA-1717 strain until 12 h after the start of the culture, but there was no difference in growth with or without Rg3 at 24 h. The minimum values of the FID index from the MIC values are shown in the combination of Rg3 with ampicillin, oxacillin, carbenicillin, cefazolin, kanamycin, and gentamicin for IID1677 and BAA-1717, indicating the synergistic activity of Rg3 with antibiotics for two strains (Table 8).

**TABLE 6:** Synergistic combination of antibiotics and ginsenosides against MRSA IID1677 strains^*a*^^,^^*b*^

Antibiotics	FIC index of antibiotics with Gs (100 µg/mL)				
	Rb1	Rg3	Re	Rg1	Rh1
Oxacillin	0.60	0.13	0.60	0.60	0.60
Ampicillin	0.60	0.35	0.60	0.60	0.60
Carbenicillin	1.60	0.35	1.60	1.60	1.10
Cefazolin	0.60	0.16	1.60	1.60	0.60
Kanamycin	0.60	0.16	0.60	0.60	0.60
Gentamicin	0.35	0.16	0.60	0.60	0.60
Tetracycline	1.10	1.10	1.10	1.10	1.10

^*a*^ The FIC index analysis for the combination of antibiotics and each ginsenoside (100 microg/mL).

^*b*^ Boxes shaded in gray are estimated as synergy.

**TABLE 7:** Synergistic combination of antibiotics and ginsenosides against MRSA BAA-1717 strain^*a*^^,^^*b*^

Antibiotics	FIC index of antibiotics with Gs (100 µg/mL)				
	Rb1	Rg3	Re	Rg1	Rh1
Oxacillin	1.10	0.35	1.10	1.10	1.10
Ampicillin	1.10	0.35	1.10	1.10	1.10
Carbenicillin	1.10	0.35	1.10	1.10	1.10
Cefazolin	1.10	0.22	1.10	1.10	1.10
Kanamycin	1.10	0.12	1.10	1.10	1.10
Gentamicin	0.35–0.60	0.16	1.10	1.10	1.10
Erythromycin	10	0.60	1.10	1.10	1.10

^*a*^ The FIC index analysis for the combination of antibiotics and each ginsenoside (100 microg/mL).

^*b*^ Boxes shaded in gray are estimated as synergy.

**TABLE 8:** Synergistic combination of antibiotics and ginsenoside Rg3 against MRSA^*a*^^,^^*b*^

Antibiotics	FIC index (min): antibiotics + Rg3	
	IID1677	BAA-1717
Oxacillin	0.04	0.27
Ampicillin	0.26	0.26
Carbenicillin	0.13	0.26
Cefazolin	0.07	0.15
Kanamycin	0.04	0.07
Gentamicin	0.07	0.05
Tetracycline	1.00	–^*c*^
Erythromycin	–^*c*^	0.52

^*a*^ The FIC index minimum (min) analysis for the combination of antibiotics and Rg3 (6.25–100 microg/mL).

^*b*^ Boxes shaded in gray are estimated as synergy.

^*c*^ (–) in the table mean not considered.

### Compound K reduces the MIC values of antibiotics against MRSA

**Fig 3:**
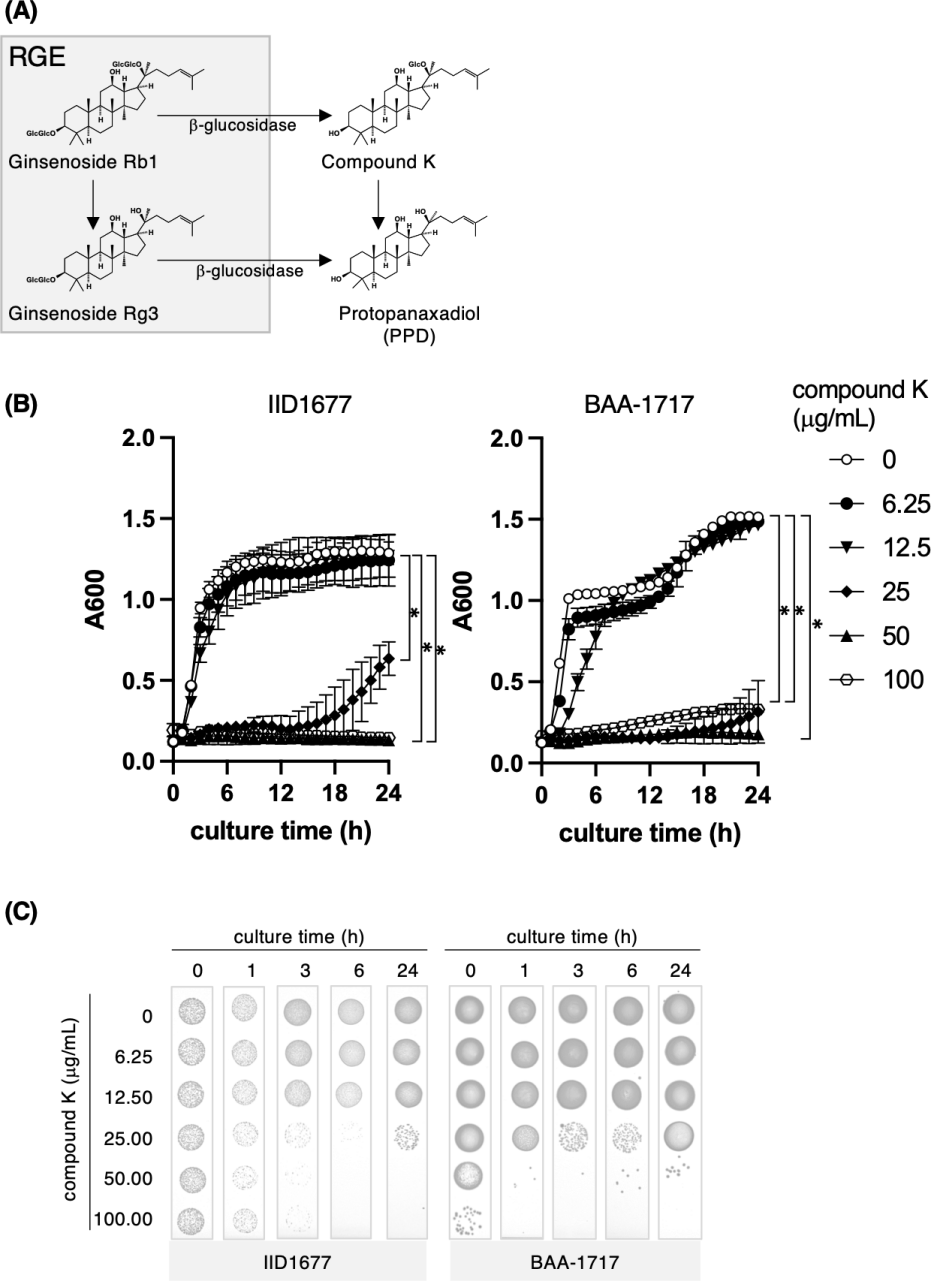
The growth of MRSA strains in the presence of compound K. (**A**) Transfer reaction of ginsenoside Rb1 and Rg3 in red ginseng extracts to compound K and/or protopanaxadiol by β-glucosidase. (**B**) The growth of the bacteria (IID1677 and BAA-1717 strains) was monitored with absorbance at 600 nm (A600) for 24 h at 37°C in the absence or presence of compound K (6.25–100 µg/mL). Before the measurement of absorbance, the plate was shaken for 5 s. One hundred microliters of suspension containing bacteria in brain heart infusion broth (OD_600_ = 0.00045) and compound K at the indicated concentrations was added per well of a 96-well plate. Values are expressed as the mean ± SD (*n* = 3) of at least three independent biological replicates. Two-way ANOVA and Tukey’s test were used for statistical analysis; *P < 0.001. (**C**) After incubating the bacteria with each concentration of compound K for 0, 1, 3, 6, and 24 h at 37°C in a 96-well plate, a 20-µL aliquot of bacterial culture that was diluted to 10^−3^ was spotted on LB agar. Bacterial growth was visually examined.

Ginsenosides Rg3 and Rb1 comprise a triterpenoid with a dammarane skeleton, and they share the PPD aglycon; however, there are differences in the sugar moieties at positions C-3 (in Rb1 and Rg3) and C-20 (in Rb1) (Fig. 2A). Despite their similar structures, only Rg3 increased the sensitivity of MRSA to antibiotics. Therefore, to better understand the relationships between structure and function, we tested the ability of ginsenoside derivatives, namely compound K, to increase the efficacy of antibiotics (Fig. 3A). Low concentration (3.125–12.5 µg/mL) of compound K showed synergistic activity with β-lactam and aminoglycoside antibiotics (Table 9). We found that more than 25 µg/mL of compound K suppressed bacterial growth for 24 h (Fig. 3B). Furthermore, compound K showed bactericidal activity against IID1677 or BAA-1717 only at 1 h after addition to the culture (Fig. 3C). These results suggest that the sugar moieties at different positions on the ginsenoside ring contribute to the bacterial activity observed.

**TABLE 9:** Synergistic combination of antibiotics and compound K against MRSA^*a*^

Antibiotics	FIC index (min): antibiotics + CK	
	IID1677	BAA-1717
Oxacillin	0.16	0.50
Ampicillin	0.38	0.25
Carbenicillin	0.56	0.31
Cefazolin	0.31	0.25
Kanamycin	0.28	0.16
Gentamicin	0.31	0.28
Tetracycline	1	–^*c*^
Erythromycin	ND^*d*^	0.50

^*a*^ The FIC index minimum (min) analysis for the combination of antibiotics and compound K (6.25–100 microg/mL).

^*b*^ Boxes shaded in gray are estimated as synergy.

^*c*^ (–), mean not concidered, because this strain is sensitive to tetracycline.

^*d*^ ND meas not determined.

## DISCUSSION

In the present study, we confirmed that RGE, which comprises more than 90% ginsenosides, acted as an antibiotic adjuvant against two laboratory strains of MRSA. Ginsenoside Rg3 present in RGE showed synergic activity with β-lactam antibiotics and aminoglycoside antibiotics for both strains. In contrast, RGE and Rg3 did not reduce resistance to fosfomycin, tetracycline, or macrolide antibiotics. Saponins, which are derived from various parts of plants (e.g., seeds, roots, and leaves), act as surfactants to induce hemolysis; however, ginsenosides extracted from the root of *Panax ginseng* contain no surfactants and thus have no inherent hemolytic activity. Thus, ginsenosides have long been used in herbal medicine, health foods, and cosmetics. Here, we compared the antibacterial activity of RGE with that of Triton X-100, a non-ionic detergent that has increased bacterial susceptibility to β-lactam antibiotics (15), because we hypothesized that RGE might have increased the permeability of the bacterial membranes. Studies suggest that Triton X-100 induces the autolysis of bacteria by increasing the release of LTA, the function of which is to inhibit autolysis (4). We confirmed that Triton X-100 increased the sensitivity of two MRSA strains to β-lactam and aminoglycoside antibiotics (Tables 2 and 3). However, whereas Triton X-100 increased the sensitivity of strain IID1677 to fosfomycin and tetracycline and the sensitivity of strain BAA-1717 to fosfomycin, RGE did not. These results suggest that the mechanism by which RGE increases the sensitivity to antibiotics is different from that used by Triton X-100. Bacterial resistance toward the antibiotic compound is the result of target modification through a mutation, decreased permeability of the bacterial cell to the antibiotics, reduction of the antibiotic influx or increase of its efflux, and deactivating processes that degrade the antibiotic such as enzymatic hydrolysis or any other chemical modification that reduces the affinity of the antibiotic for its target (16). In the present study, it is unclear how Rg3 reduced the MIC values of antibiotics. However, Rg3 might be involved in processes other than the renewal of bacterial cell permeability to the antibiotics. The lack of synergistic activity of fosfomycin with RGE may provide a clue to the answer. RGE did not affect the growth of the IID1677 strain (Fig. 1A) and methicillin-sensitive *S. aureus* JCM2151 (data not shown), while RGE enhanced the early growth of BAA-1717 strain. In contrast, Rg3 did not enhance the growth of BAA-1717. Therefore, the fact that RGE promoted bacterial growth indicates that it was at least an interaction with something other than Rg3 (Fig. 2C). The growth of BAA-1717 was biphasic, but the growth of IID1677 was not. The two-phase growth curves of *S. aureus* (17) and *Escherichia coli* (18) may differ in the main carbon source of ATP production. The biphasic growth curves of the BAA-1717 strain in brain heart infusion (BHI) broth did not show when the bacteria were cultured in LB broth. Something present in RGE might have changed the biphasic growth curve of BAA-1717.

In addition to the spread of HA-MRSA and CA-MRSA isolated from humans, livestock-associated MRSA (LA-MRSA) is an emerging problem worldwide (19). RGE showed 88.2% synergistic efficacy against kanamycin-resistant CA-MRSA (15 of 17 strains) (Table 4) and 71.4% against ampicillin-resistant LA-MRSA (five of seven strains) (Table 5). In particular, the MIC value of kanamycin, which was highest against CA-MRSA (No. 8), decreased by 0.063-fold (from 10,000 to 625 μg/mL) in the presence of RGE. Thus, RGE increases the sensitivity of MRSA, which causes a variety of human and animal infections, to kanamycin and ampicillin.

Of the five ginsenosides tested, only Rg3 increased the sensitivity of MRSA to β-lactam and aminoglycoside antibiotics. Rg3 is a minor ginsenoside component of RGE, accounting for about 1% of the total RGE weight (6, 7). Here, we found that 12.5–25 µg/mL of Rg3 increased the sensitivity of MRSA to antibiotics to the same extent as 1 mg/mL of RGE, suggesting that Rg3 is a potent antibacterial component of RGE. Furthermore, we found that compound K (12.5–25 µg/mL) showed similar results to Rg3. Both Rg3 and compound K contain the PPD aglycon and either two or one glucose moieties (8). By contrast, ginsenoside Rb1, which also contains the PPD aglycon, did not reduce the resistance of MRSA to antibiotics. Therefore, the number of sugar moieties may be an important factor that determines bactericidal effects. We found that compound K exerted bactericidal activity at more than 25 µg/mL (Fig. 3B and C). It is unclear why compound K has bactericidal activity. Future studies will attempt to unravel the underlying mechanism. Neither Rg3 nor compound K are “natural” chemicals present in unprocessed *Panax ginseng*. Rg3 is a ginsenoside degraded from other abundant ginsenosides during heat processing of fresh ginseng to manufacture red ginseng (12). In the present study, compound K was synthesized from butanol extracts (crude extracts) of red ginseng by pectinase G. By contrast, compound K is known to be derived from ginsenoside Rb1 via the activity of β-glucosidases released by gut microbes (20, 21) (Fig. 3A). Therefore, compound K is enzymatically synthesized from ginseng extracts; for example, by β-galactosidase from *Aspergillus oryzae,* lactase from *Penicillium* sp. (13), and β-D-glucosidase from *Lactococcus lactis* (22). However, it is unknown whether compound K was methanolized from RGE by *S. aureus*. Compound K (25 µg/mL) showed bactericidal activity at low amounts of bacteria (3 or 6 h after incubation), but the molecule count of compound K was insufficient at high bacteria levels (>6 h after incubation) (Fig. 3B). In contrast, compound K of more than 50 µg/mL has shown bactericidal activity at least 1 h after incubation of bacteria and persisted for 24 h, suggesting that it might be an antibiotic for MRSA.

In the previous study, RGE inhibited the phosphorylation of p70 ribosomal S6 kinase in the ears of mice with AD, resulting in the suppression of skin inflammation (5). *S. aureus* is a commensal bacterium that colonizes the skin of one-third of humans, and multidrug-resistant *S. aureus*, called CA-MRSA, has recently increased among healthy individuals. Thus, RGE and Rg3 might exhibit antimicrobial activity against MRSA in skin with AD to use in combination with low concentrations of antimicrobials. A previous study reports that compound K exerts anti-inflammatory activity by regulating the NF-κB pathway in macrophages, which was stimulated by Toll-like receptor ligands (23). Thus, Rg3 and compound K might be universal agents that possess both bactericidal activity and anti-inflammatory activity against *S. aureus*-mediated diseases.

In conclusion, ginsenoside Rg3 and nonnatural compound K, derived from major ginsenosides in *Panax ginseng*, are potential new antibiotic adjuvants that are associated with conventional antibiotics for MRSA.

## MATERIALS AND METHODS

### Materials

Triton X-100 was purchased from Fujifilm (Osaka, Japan). Brain heart infusion broth was purchased from Becton Dickinson (Tokyo, Japan). All antibiotics used in this study were purchased from Wako (Osaka, Japan).

### Purification of RGE and synthesis of compound K

Red ginseng produced from 6-year-old ginseng root was obtained from Ohki Pharmaceutical Co., Ltd. (Tokyo, Japan). Five hundred grams of red ginseng was crushed and refluxed for 2 h (twice) in 5 L of 70% methanol. The filtrate was evaporated to dryness under reduced pressure to yield a brownish extract. The extract (RGE) was dissolved in methanol and applied to a LiChroprep Rp-18 chromatography column (Merck Millipore, Tokyo, Japan) run with a water-methanol system to obtain the saponin fraction (12). Ginsenosides (Rb1, Rg3, Re, Rg1, and Rh1) were prepared from RGE by high-performance liquid chromatography (12). Approximately 3-10 g of RGE and 0.3 g of Rg3 were extracted from 1 kg of red ginseng used in this study. Briefly, butanol extracts of red ginseng were separated on a LiChroprep Rp-18 column, and the PPD fractions were eluted with 40% methanol after washing with 0%–20% methanol. After drying the PPD fractions in a rotary evaporator, the resulting PPD powder (5.0 g) was dissolved in water (500 mL), and the pH was adjusted to 4.0 with hydrochloric acid. After the addition of pectinase G (5.0 g) (Amano enzyme, Tokyo, Japan), the PPD was incubated at 50°C for 72 h, filtered through glass wool (GL Sciences, Tokyo, Japan), and concentrated in a rotary evaporator. The concentrated PPD extract was then redissolved in methanol, and the sugar (insoluble fraction) was removed. The PPD extract was applied to a LiChroprep Rp-18 column, and the compound K fraction was eluted using 80% methanol. After concentration in a rotary evaporator, compound K was purified on a silica gel 60 F254 column (Merck Millipore) using a mobile phase comprising 20:1 chloroform:methanol. Approximately, 0.03 grams of compound K was prepared from 1 kg of red ginseng.

### Bacteria strains and growth conditions

The MRSA strain IID1677 was provided by the Institute of Medical Science, University of Tokyo, through the National Bioresource Project of the MEXT, Japan. Strain BAA-1717 [TCH1516 (USA300-HOU)] was provided by the ATCC. MRSA (17 strains) isolated from healthy volunteers was provided by Professor Komatsuzawa (Hiroshima University), and MRSA (seven strains) isolated from bovine mastitis lesions was provided by Dr. Mandokoro (Kyoto Prefectural Chutan Livestock Health Hygiene). *S. aureus* strains were cultured at 37°C in BHI broth. Bacterial growth during static incubation was monitored by measuring the absorbance at 600 nm (Spectra Max i3X; Molecular devices, Japan) upon culture in a 96-well bottom round plate (TPP, Switzerland).

### Measurement of minimal inhibitory concentrations

*S. aureus* was suspended in BHI broth in a well of a 96-well plate (TPP) and then mixed with RGE (1–5,000 μg/mL), five ginsenosides (Rb1, Rg3, Re, Rg1, and Rh1; 100 µg/mL), or Triton X-100 (0.0025%–0.02%) with antibiotics (oxacillin: 3.91–1,000 µg/mL, ampicillin: 7.81–2,000 µg/mL, carbenicillin: 3.91–1,000, cefazolin: 7.81–1,000 µg/mL, fosfomycin: 3.15–500 µg/mL, kanamycin: 78.1–20,000 µg/mL, gentamicin: 0.0391–10 µg/mL, tetracycline: 1.56–200 µg/mL, erythromycin: 1.95–500 µg/mL, levofloxacin: 0.0391–10 µg/mL, and vancomycin: 0.0391–10 µg/mL) at the indicated concentrations. The bacteria were statically cultured at 37°C for 20–24 h. The MIC values were visually defined as the lowest concentration of antibiotic that inhibited bacterial growth. When it was difficult to judge, ATP levels in bacterial culture were measured by chemiluminescence using Bac Titer Gro (Promega, Tokyo, Japan). Ethanol used to dissolve erythromycin was at a final concentration of 0.5% in the bacterial culture medium. Other antibiotics were dissolved in sterile water. Methanol used to dissolve RGE and ginsenosides as a vehicle was at a final concentration of 0.5% in the bacterial culture medium. Thus, 0.5% ethanol or 0.5% methanol was used as a control. All experiments were performed three or more times from three or more experiments with at least three independent biological replicates.

### FIC and FIC index calculation

Fractional inhibitory concentration values were calculated using the following formula:


$$\frac{MIC_{A/B}}{MIC_{A/0}}+\;\frac{MIC_{B/A}}{MIC_{B/0}}=FIC_{A}+FIC_{B}=FIC\;index$$

MIC_*A*/0_ is the MIC value of *A* alone, and MIC_*B*/0_ is the MIC value of *B* alone. MIC_*A/B*_ is the MIC value of *A* in the presence of some concentration of *B*, and MIC_*B/A*_ is the MIC value of *B* in the presence of some concentration of *A*. Either FIC_*A*_ or FIC_*B*_ corresponds to the fractional MIC for *A* or *B*. The sum of these values for each individual data point is the FIC index. After deciding the MIC values of antibiotics in each concentration of RGE, the FIC index was calculated in each concentration of RGE. The minimum FIC index value over all data points is the FIC index (min). FIC index (min)＜0.5 is indicative of synergy, and the values between 0.5 and 4.0 are indicative of no synergy. The higher concentration of RGE (10 mg/mL) did not have bactericidal activity, resulting in a MIC of RGE > 10 mg/mL. The Rg3 (1 mg/mL) did not have bactericidal activity, resulting in a MIC of Rg3 > 1 mg/mL. The higher concentration of tTriton X-100 (2.0%) did not have bactericidal activity, resulting in a MIC of Triton X-100 > 2.0%.

### Bactericidal activity

*S. aureus* suspended in BHI broth was mixed with ginsenosides at different concentrations in a well of a 96-well plate. After incubation at 37°C for 24 h, the bacterial culture was appropriately diluted from 10 to 10^10^ in BHI broth and a 20-µL aliquot was spotted onto LB agar. After incubation at 37°C for 24 h, bacterial growth was examined visually.

### Thin layer chromatography

RGE and ginsenosides were dissolved in methanol and spotted onto a silica gel 60 F254 TLC plate (Millipore, Tokyo, Japan). The thin layer chromatography (TLC) plate was then placed into a chamber saturated with mobile phase (6.5:3.5:1 chloroform:methanol:water). After 30 min, the TLC plate was sprayed with 6.3% sulfuric acid containing 1.01% cerium (IV) sulfate tetrahydrate. The developed spots were detected after heating to 110°C for 10 min.

### Statistical analysis

Experiments were performed three or more times. All data are presented as the mean ± SD of at least three independent biological replicates.

## References

[1] Lee AS, de Lencastre H, Garau J, Kluytmans J, Malhotra-Kumar S, Peschel A, Harbarth S. 2018. Methicillin-resistant Staphylococcus aureus. Nat Rev Dis Primers 4:18033. doi:10.1038/nrdp.2018.33 29849094

[2] Barbieri R, Coppo E, Marchese A, Daglia M, Sobarzo-Sánchez E, Nabavi SF, Nabavi SM. 2017. Phytochemicals for human disease: an update on plant-derived compounds antibacterial activity. Microbiol Res 196:44–68. doi:10.1016/j.micres.2016.12.003 28164790

[3] Komatsuzawa H, Sugai M, Shirai C, Suzuki J, Hiramatsu K, Suginaka H. 1995. Triton X-100 alters the resistance level of methicillin-resistant Staphylococcus aureus to oxacillin. FEMS Microbiol Lett 134:209–212. doi:10.1111/j.1574-6968.1995.tb07939.x 8586269

[4] Ohta K, Komatsuzawa H, Sugai M, Suginaka H. 2000. Triton X-100-induced lipoteichoic acid release is correlated with the methicillin resistance in Staphylococcus aureus. FEMS Microbiol Lett 182:77–79. doi:10.1111/j.1574-6968.2000.tb08877.x 10612735

[5] Lee SM, Bae BS, Park HW, Ahn NG, Cho BG, Cho YL, Kwak YS. 2015. Characterization of Korean red ginseng (Panax ginseng Meyer): history, preparation method, and chemical composition. J Ginseng Res 39:384–391. doi:10.1016/j.jgr.2015.04.009 26869832 PMC4593794

[6] Yoon SJ, Kim SK, Lee NY, Choi YR, Kim HS, Gupta H, Youn GS, Sung H, Shin MJ, Suk KT. 2021. Effect of Korean red ginseng on metabolic syndrome. J Ginseng Res 45:380–389. doi:10.1016/j.jgr.2020.11.002 34025131 PMC8134847

[7] Samukawa K, Izumi Y, Shiota M, Nakao T, Osada-Oka M, Miura K, Iwao H. 2012. Red ginseng inhibits scratching behavior associated with atopic dermatitis in experimental animal models. J Pharmacol Sci 118:391–400. doi:10.1254/jphs.11182fp 22382656

[8] Han MJ, Kim DH. 2020. Effects of red and fermented ginseng and ginsenosides on allergic disorders. Biomolecules 10:1–17. doi:10.3390/biom10040634 PMC7226199 32326081

[9] Chen W, Balan P, Popovich DG. 2019. Review of ginseng anti-diabetic studies. Molecules 24:4501. doi:10.3390/molecules24244501 31835292 PMC6943541

[10] Chen W, Balan P, Popovich DG. 2019. Analysis of ginsenoside content (Panax ginseng) from different regions. Molecules 24:3491. doi:10.3390/molecules24193491 31561496 PMC6803836

[11] Osada-Oka M, Hirai S, Izumi Y, Misumi K, Samukawa K, Tomita S, Miura K, Minamiyama Y, Iwao H. 2018. Red ginseng extracts attenuate skin inflammation in atopic dermatitis through P70 ribosomal protein S6 kinase activation. J Pharmacol Sci 136:9–15. doi:10.1016/j.jphs.2017.11.002 29274665

[12] Samukawa K, Yamashita H, Matsuda H, Kubo M. 1995. Simultaneous analysis of saponins in ginseng radix by high performance liquid chromatography. Chem Pharm Bull. 43:137–141. doi:10.1248/cpb.43.137

[13] Raychaudhuri D, Chatterjee AN. 1985. Use of resistant mutants to study the interaction of Triton X-100 with Staphylococcus aureus. J Bacteriol 164:1337–1349. doi:10.1128/jb.164.3.1337-1349.1985 2866176 PMC219335

[14] Komatsuzawa H, Suzuki J, Sugai M, Miyake Y, Suginaka H. 1994. The effect of Triton X-100 on the in-vitro susceptibility of methicillin-resistant Staphylococcus aureus to oxacillin. J Antimicrob Chemother 34:885–897. doi:10.1093/jac/34.6.885 7730232

[15] Komatsuzawa H, Sugai M, Ohta K, Fujiwara T, Nakashima S, Suzuki J, Lee CY, Suginaka H. 1997. Cloning and characterization of the fret gene which affects the methicillin resistance level and autolysis in the presence of Triton X-100 in methicillin-resistant Staphylococcus aureus. Antimicrob Agents Chemother 41:2355–2361. doi:10.1128/AAC.41.11.2355 9371333 PMC164128

[16] Douafer H, Andrieu V, Phanstiel O, Brunel JM. 2019. Antibiotic adjuvants: make antibiotics great again! J Med Chem 62:8665–8681. doi:10.1021/acs.jmedchem.8b01781 31063379

[17] Vitko NP, Grosser MR, Khatri D, Lance TR, Richardson AR. 2016. Expanded glucose import capability affords Staphylococcus aureus optimized glycolytic flux during infection. mBio 7:e0029616. doi:10.1128/mBio.00296-16 PMC4916373 27329749

[18] Kimata K, Takahashi H, Inada T, Postma P, Aiba H. 1997. cAMP receptor protein-cAMP plays a crucial role in glucose-lactose diauxie by activating the major glucose transporter gene in Escherichia coli. Proc Natl Acad Sci U S A 94:12914–12919. doi:10.1073/pnas.94.24.12914 9371775 PMC24238

[19] Anjum MF, Marco-Jimenez F, Duncan D, Marín C, Smith RP, Evans SJ. 2019. Livestock-associated methicillin-resistant Staphylococcus aureus from animals and animal products in the UK. Front Microbiol 10:2136. doi:10.3389/fmicb.2019.02136 31572341 PMC6751287

[20] Hasegawa H, Sung JH, Matsumiya S, Uchiyama M. 1996. Main ginseng saponin metabolites formed by intestinal bacteria. Planta Med 62:453–457. doi:10.1055/s-2006-957938 8923812

[21] Akao T, Kida H, Kanaoka M, Hattori M, Kobashi K. 1998. Intestinal bacterial hydrolysis is required for the appearance of compound K in rat plasma after oral administration of ginsenoside Rb_1_ from Panax ginseng. J Pharm Pharmacol 50:1155–1160. doi:10.1111/j.2042-7158.1998.tb03327.x 9821663

[22] Li L, Lee SJ, Yuan QP, Im WT, Kim SC, Han NS. 2018. Production of bioactive ginsenoside Rg3(S) and compound K using recombinant Lactococcus lactis. J Ginseng Res 42:412–418. doi:10.1016/j.jgr.2017.04.007 30337801 PMC6187048

[23] Choi S, Kim T. 2023. Compound K - an immunomodulator of macrophages in inflammation. Life Sci 323:121700. doi:10.1016/j.lfs.2023.121700 37068708

